# Suitability of Test Procedures for Determining the Compatibility of Seal Materials with Ionic Hydraulic Fluids

**DOI:** 10.3390/polym16182551

**Published:** 2024-09-10

**Authors:** Darko Lovrec, Vito Tič

**Affiliations:** Faculty of Mechanical Engineering, University of Maribor, Smetanova 17, 2000 Maribor, Slovenia; vito.tic@um.si

**Keywords:** ionic hydraulic fluids, seal material compatibility, static and dynamic test, comparison of results, differences, test suitability

## Abstract

The compatibility of seal materials with the working fluid is crucial for the flawless, energy-saving, environmentally sustainable, and safe operation of any technical system. This is especially true for hydraulic systems operating under high operating pressure. The problem of materials compatibility comes into play when either a new type of seal material or a new type of fluid comes into use. The paper discusses the research findings regarding material compatibility testing of new high-tech ionic hydraulic fluids with commonly used seal materials. Due to the completely different chemical composition of these new fluids compared to the classical mineral-based oil, for these fluids, there are no standardized testing procedures. In these cases, we can only lean on the Standards that apply to classical fluids, which can lead to incorrect results. In the forefront of the paper is the discrepancy between the results obtained by the standardized test, and the test under real operating conditions. FKM, an excellent material for seals, proved to be the most suitable in the case of using ionic hydraulic fluid, according to a standardized test. However, it failed in the comparison test under real operating conditions, as the cylinder leaked. NBR seals proved to be a better solution.

## 1. Introduction

Hydraulic fluid is a key component of any hydraulic system, as it must meet many functional, technical, and economical requirements. At the same time, the environmental suitability of the fluid is increasingly at the forefront, as most working machines, from construction, agriculture, forestry, and other vehicles and devices, operate in nature. In addition, hydraulic systems are becoming smaller and loaded with ever-increasing operating pressures. Apart from that, they are increasingly operating in harsher climatic operating conditions. With classic hydraulic fluids, we can no longer meet all the mentioned requirements. Completely new high-tech ionic hydraulic fluids, HIL, began to be developed and used as a result. In addition to excellent physical and chemical material properties, they have other excellent properties: they are non-flammable, and, at the same time, environmentally friendly. Due to all the mentioned features, HILs are in use, especially in areas where conventional hydraulic fluids cannot solve all the requirements satisfactorily. They are particularly suitable for operating devices in harsh operating conditions, e.g., at very low or high temperatures, in vulnerable environments, in remote locations, and elsewhere. More information about ionic liquids and the excellent physicochemical properties of ionic hydraulic fluids, as well as application areas, can be found in various sources, e.g., [1,2,3,4,5,6,7,8,9,10].

Ionic hydraulic fluids are currently still considered alternative hydraulic fluids, such as using water or a water-based hydraulic fluid, where it is necessary to use appropriate component materials or special coatings, which limits and makes their use more expensive [11,12,13,14]. With ionic hydraulic fluids, there are no restrictions or limitations regarding equipment, as is the case with other hydraulic fluids (e.g., HFC-type fluid or water). Thus, for example, in the case of using a classic mineral hydraulic oil, without any changes or adaptations of the equipment, you can just replace it with ionic hydraulic fluid.

In addition to the general environmental acceptability of the hydraulic fluid itself, the flawless condition of the hydraulic components themselves must also be ensured (e.g., [15,16,17]). Here, the problem of leaking hydraulic components is always potentially present. This is not only related closely to the correct loading of the component and the cleanliness of the hydraulic fluid, which affects the wear of the installed seals directly, but also to the compatibility of the fluid with the seal material. From this point of view, seals are key components that ensure the long-lasting, flawless, energy-saving, and economical, as well as environmentally friendly, operation of the entire hydraulic system. Usually, there is not just one seal inside a hydraulic component, e.g., a simple O-ring seal, but a set of seals and guide rings that, mutually, ensure the flawless operation of the component.

**Figure 1:**
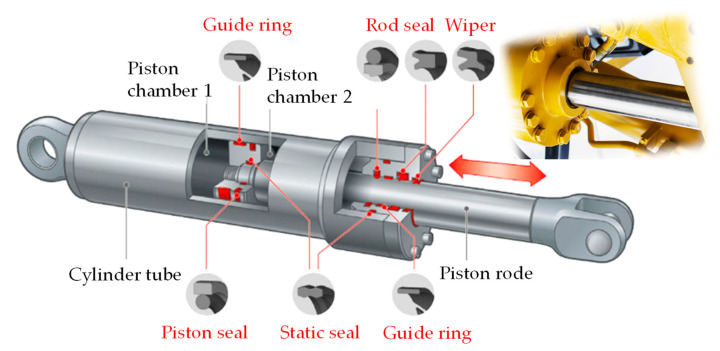
Variety of hydraulic cylinder seals.

Hydraulic cylinders represent such an example, as the most frequently used very statically and dynamically loaded hydraulic component, wherein a whole set of seals is installed for the following: for internal and external sealing of the piston and piston rod, for wiping the impurities from the piston rod, and for guiding and keeping the piston rod in the appropriate position (guide rings). Therefore, the problem of seals in hydraulic cylinders is the subject of many different studies (e.g., [18,19,20,21,22]). Also, the shapes of the seals themselves and the materials used for the seals are very different, depending on the intended use of the cylinder and the load. Figure 1 shows the different types, shapes, and locations of seals in the case of a conventional hydraulic cylinder, as represented by several specialist suppliers of hydraulic seal sets (e.g., [23,24,25,26]).

Seal manufacturers are aware of the extreme importance and problems of seals, which is why they invest a lot of effort in new quality seal materials and suitable seal shapes. Modern hydraulic components incorporate several materials, choosing them based on the type of hydraulic fluid, purpose, and operating parameters. Thus, in practice, seals are made of polyurethane (HPU), elastomers such as nitrile (NBR), fluorocarbon, elastomers based on fluor rubber (FKM), ethylene propylene (EPR, EPDM), chloroprene (CR), urethane (AU, EU), polyacetal (POM), polyamide (PA), polytetrafluoroethylene (PTFE), polyethylene (PE), polyetheretherketone (PEEK), and up to organic thermoplastic polymer and silicone (MVQ). Certain seal materials have been known for quite some time (e.g., NBR and EPDM since 1955). However, new materials keep appearing, which is why seals are also the subject of constant development. In the case of new materials, the compatibility of the sealing material with the specific liquid must be checked.

## 2. Seal Material Compatibility Testing

Before using a certain type of seal material with a certain fluid, it is necessary to check the mutual compatibility of both materials. The importance of the compatibility of seal materials with different liquids is evidenced by many different studies, e.g., [27,28,29,30,31,32].

Most of the knowledge related to compatibility refers to the known and commonly used hydraulic fluids, especially the most used hydraulic mineral oil. Thus, compatibility testing is usually performed, based on the detailed prescribed test procedure for well-known fluid (i.e., mineral oil) given in various Standards (e.g., ASTM D1414, ASTM D471, ASTM D4289, ASTM D6546, ASTM D2240, etc. [33,34,35,36]). The basic characteristics of the testing procedures or Standards are also summarized in the professional literature, e.g., [37], including comments and reference values for a specific property change.

When mentioning these Standards, it should be emphasized that there are no Standards for other types of fluids, so we have no choice other than to lean on the available ones. In the case of testing the compatibility of HIL, as a completely new type of fluid, we used the mentioned Standards as a starting point.

In the case of the mentioned Standards, it is a static method of compatibility testing with thermal loading for a prescribed number of hours and for a certain form of the test sample, whereby the test sample is evaluated after each phase of thermal loading. The test samples are analyzed before testing and after 24, 70, 100, 250, 500, or 1000 h. A test typically runs at 90 °C, which corresponds to the approximate operating temperature inside the hydraulic component. The shape of the seal test sample must match the shape of the seals used. Thus, shapes close to the actual sizes of O-rings are used for testing, e.g., 2-021, -120, -214, or -320 (according to Standard AS568 [38]). In our case, we used a ring seal form with a square cross-section, which, in terms of shape, corresponds to today’s variants of seals suitable for higher operating pressures (for more details see [28,39]).

Swelling and shrinkage of the test sample, as the first significant changes, are determined by weighing the mass of the sample, which is carried out before and after each stage of testing. According to the procedure, it is necessary to weigh the initial mass of the sample in air and then immerse it in water at room temperature. This is followed by heating the sample in the test fluid, at a specified temperature and duration for a specified time, and then, after cooling, the sample is reweighed in air and water.

The shrinkage test is performed similarly to the swelling test and has a similar range of measurements, except that, in this case, the mass is also determined by the specimen in air and in water after drying out. After each phase of testing, the change in volume is calculated according to the corresponding equation given in the Standard. More details about the testing process itself and the evaluation of the results are available in the mentioned Standards and in the related literature [39].

The occurrence of swelling or shrinkage is the first indicator of seal material compatibility. In addition, it is necessary to determine the change in hardness, and possibly a change in the mechanical properties of the sealing material, as provided by the Standard. Based on the given recommended limits of individual changes, we can judge the suitability of the seal material based on its compatibility with a certain fluid [40].

### 2.1. Compatibility of Ionic Hydraulic Fluids with Hydraulic Seals’ Materials

Among the various ionic hydraulic fluids, in this case, the emphasis will be on HIL B2002a^TM^ (a dialkylimidazolium-based ionic liquid, produced by the company proionic GmbH). As an ionic hydraulic fluid, HIL B2002a^TM^ has proven itself in use, due to its excellent physicochemical properties, and has been used in the metallurgical industry for some time. The selection process of the appropriate ionic hydraulic fluid based on physicochemical properties is described in more detail in [10]. Table 1 shows the results of compatibility testing with HIL B2002a^TM^, only for those seal materials that have passed all the time phases of thermal loading, including 500 h.

For comparison, a classical mineral-based hydraulic oil of the HLP type classification (according to DIN 51524 part 2) was used. The oil contains selected additives to improve aging resistance, wear and corrosion protection, and EP properties. The HLP type of hydraulic oil is also most often used in hydraulic systems.

Eight of the most used seal materials in hydraulics were tested in the considered case: FKM, POM, HPU Franc, NBR, HPU USI, EPDM, MVQ, and PTFE I. Certain seal materials were, namely, already degraded in a shorter time and excluded from further testing (e.g., HPU Franc). To compare the results with classic hydraulic fluid, the compatibility test results also include hydraulic mineral oil type HLP.

The results shown refer to 70, 250, and 500 h of testing the three most important changes in seal materials: Volume swell, Shrinkage, and Hardness. These three parameters are a good starting point for the first conclusions regarding the seal material compatibility. For an easier interpretation of the results, the reference values of the individual changes are given at the beginning of the Table, which are given in the professional literature for the case of using mineral oil. For easier transparency, the reference average values for oil and values that do not deviate much from the reference values are shown in black (for both, oil and HIL), values that are better than the reference values (e.g., smaller changes from the reference, or no changes) are in green, and larger deviations are in red.

As can be seen from the results, even in the case of the tested classical HLP-type mineral oil, greater or smaller deviations of changes from the reference values occur. The latter results from the fact that the values given as the reference refer to the average values for this type of oil (without known details regarding the additive package used). Based on this, we can also evaluate the compatibility of the most used type of hydraulic oil with the seal materials, which is somehow considered the starting point for all further comparisons.

### 2.2. Additional Aspects of Testing

When evaluating the adequacy of the seal material, several aspects must be considered: the size of the deviation from the reference value that applies to mineral oils, the assessment of whether it is a small or large deviation, the deviation trend, and also the type of seals’ material, which manufacturers, based on their experience, most often incorporate into their components. Regarding the deviation trend, it is necessary to consider whether the individual parameter deteriorates with the duration of the test, or whether the value remains unchanged after the initial degradation, which is also an important aspect regarding the deterioration of the sealing performance. Thus, e.g., in the case of the FKM material, the values deteriorated already in the case of mineral-based oil, while, in the case of HIL B2002a^TM^, there were almost no changes, or they deteriorated minimally with the time of testing.

Apart from the mentioned changes, the color of the FKM material also changed during thermal stress, from dark brown to almost black in the next test stages. Table 2 shows the change in color after different stages of thermal loading.

According to the given numerical values in Table 1, PTFE is the most suitable material, both for the case of using mineral hydraulic oils, as well as for the considered HIL B2002a^TM^, since practically no changes occurred. This confirms that PTFE is a synthetic fluoropolymer with excellent chemical, tribological, and thermal properties, practically resistant to all hydraulic fluids, appropriate for dynamic applications, and operating pressures at least up to 400 bars. The same applies to the POM and EPDM. However, due to their hardness, all three mentioned materials are in practice more suitable for use as guide rings than for elastic dynamically loaded seals, such as those that are found in hydraulic cylinders. Considering the results of the standardized compatibility testing method and all other aspects mentioned, FKM and NBR are thus the most suitable materials for dynamic seals, with FKM being a slightly more suitable choice in view of the changed values.

## 3. Compatibility of Seal Materials under Real Operating Conditions

When it comes to a completely new type of hydraulic fluid, such as ionic hydraulic fluids, conventional, albeit standardized, tests are not sufficient, as they do not necessarily provide comparable results to performance under real operating conditions. Here, for example, the influence of high operating pressure, dynamic loading of seals, change in the direction of movement, additional influence of friction, and more, appears.

The Standard tests refer to well-known fluids (perhaps with a slightly changed composition, e.g., due to a changed type of additives), but, in the case of a different fluid, the results may deviate (in certain cases very much). The latter can lead to inconveniences or even catastrophic consequences under real operating conditions. In such cases, it is necessary to check the compatibility of the materials under real operating conditions. The results of the static test serve as a good starting point for the use of a suitable seal material.

### 3.1. Comprehensive Durability Testing of Hydraulic Components with New Hydraulic Fluids

In the field of hydraulic drive technology, different approaches to testing are used for the purposes of component development, suitability, and durability of various materials, the mutual influence of the hydraulic medium on individual materials, and the energy aspect of operation. It depends on which aspect is the focus of each test. These are usually standardized or dedicated tests of longer or shorter duration, with more demanding operating conditions and with a constant or changing load on the system and components (e.g., [41,42,43,44,45,46]).

In cases where it is a question of the compatibility of an otherwise known sealing material with a completely new type of hydraulic fluid, as in our case, it makes sense to check this property in various ways. It is not necessary that standardized testing procedures lead to credible conclusions. In such cases, it is necessary to use another approach for comparison. It certainly makes sense to check the material compatibility also under actual operating conditions, under realistic dynamically changing pressures and actual friction between the seal and the moving parts of the cylinder. The latter can only be checked on a purpose-built test device that operates under realistic, or, even better, slightly stricter operating conditions.

**Figure 2:**
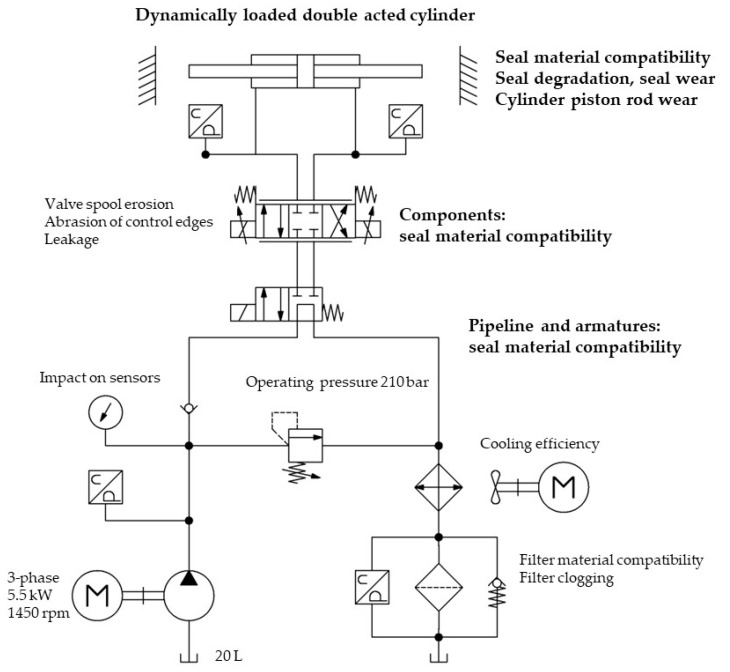
Test device for long-term testing of the impact of new hydraulic fluids on system components and overall system behavior with points of interest [47].

The compatibility test and the behavior of the seals in a dynamically loaded hydraulic cylinder is one of the focuses of the comprehensive test under real conditions. As part of the comprehensive test, we can monitor several changes and parameters at the same time, including possible changes to the static seals in the pipeline connections, in the pump, and in other components of the hydraulic system. The test device used for the long-term comparative testing of HIL vs. mineral oil is shown in Figure 2. More details regarding the test device design and the test procedure can be found in [47].

The purpose-designed test device provides insight into the wear of all vital components of a conventional hydraulic pump device (pump, various valves, and actuators). In addition to the wear of the components, it also allows determining the influence of the tested fluid on various materials used within all components of the hydraulic system. All mentioned points of interest are monitored either online or offline. In the considered case, the focus was on the compatibility of the seal material with HIL B2002a^TM^ under real operating conditions.

Particular attention was paid to the concept of a very rigorous cyclic loading of the hydraulic components and of the whole system. Cyclic loading was carried out via a specially designed loading unit, with a symmetric double-acting hydraulic cylinder as the actuator controlled by a proportional directional valve and with a mechanical backrest (end-plates for applying the load) on each side.

**Figure 3:**
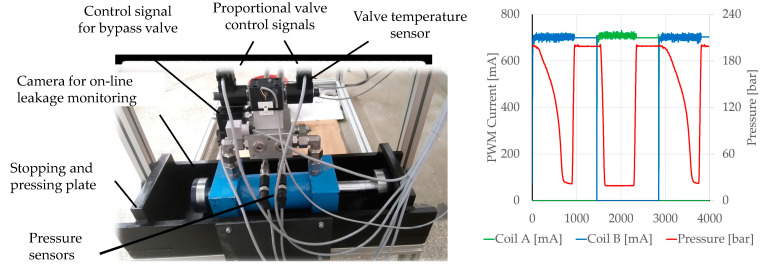
Load unit with sensors (**left**) and valve control signal profile (**right**).

The test lasts according to a predetermined number of load cycles, e.g., one million cycles (which corresponds to several weeks of continuous operation). Each cycle consists of moving the cylinder toward one side of the loading unit, after reaching a predetermined pressure (corresponding to the pressing force on the loading unit housing), and only holding this for a certain time, followed by a movement to the opposite side. Figure 3 shows a load unit equipped with sensors for online monitoring of the load unit and the valve.

The cycle time was determined based on the response of the proportional control valve and the time it took for the cylinder piston to move from one side of the load unit to the other and switch the movement back to the initial point. The duration of the entire cycle, movement in both directions, was set to 2.8 s, and the length of the testing was set to reach the one million completed cycles (or run to failure). The latter proved to be suitable, based on all previous tests of this type with a known fluid (mineral oil), where all the vital components (valves, pumps, and moving parts of the cylinder including seals) were replaced with new ones. During this time, all the changes appeared as a result of component degradation or failure.

The proportional valve control signal change causes a change in operational pressure, which varies between 5 bar and 210 bar, with a total period of two changes in 2.8 s. The pulse variation in the pump pressure at a frequency of approximately 1 Hz is achieved in this way. All the built-in seals were also dynamically and cyclically loaded in this way.

### 3.2. Types of Seal Materials in the Tested Components

Several dilemmas arise when deciding on the appropriate seal material. One of these is that manufacturers install only one type of seal material in their components, as, only in certain cases, is it possible to choose between two materials. Different manufacturers do not always use the same type of material, but rather different ones, depending on the experience of the individual manufacturer. Additionally, manufacturers decide on the type that has proven to be the most suitable for their component, usually for mineral oil. So, different seals made of different materials are used in different hydraulic components, whether it is seals in the pump, cylinder, valve, connections, or sensors or seals on the hydraulic tank, seals on the cooling unit, etc.

Thus, we encounter a variety of materials in the components that make up the hydraulic system, and these are generally suitable for mineral oil. For other types of hydraulic fluids, e.g., fire-resistant HFC types (water- and glycol-based) or synthetic ones (HFD types), the intended use of non-oil fluids must be specified when ordering the component. However, in the case of the use of a special fluid, it is important to consult the component manufacturer to install the seal of the appropriate material. This is possible only in cases when the compatibility of materials is known. In the case of completely new fluids, such as HIL, the question of compatibility remains open.

It is the same situation in the case of HIL, except that they have not been used on real hydraulic systems before the compatibility test, so there is no relevant information.

An additional aspect to keep in mind is that we follow an important practical goal: replacing mineral hydraulic oil with ionic hydraulic fluid, without requiring changes on the components, especially on the seals. This added an additional challenge to the problem of HIL compatibility with different seal materials.

Apart from dynamic seals in hydraulic cylinders, in other components, there are mainly static seals, which ensure sealing between two stationary parts. These seals are not dynamically loaded, so, the test focus is more on dynamically loaded hydraulic cylinder seals. For these seals, it is necessary to choose materials that, in addition to thermal stability, tolerate dynamic pressure changes and frictional loads well, namely minimal wear of the seal during long-term use. Even under harsher operating conditions, the seals must ensure the perfect functioning of the cylinder, and, thus, have a long service life.

By conforming to the complex and linked problem of selecting a seal material according to the fluid used, it is especially important to focus on the seal material of the hydraulic cylinder. In the case of hydraulic cylinders, a wide variety of materials are also used for dynamic seals. For static seals in other components, the offered range of seal materials is smaller. In view of unifying the material for all seals of all components, it would certainly be best to use the same type of material in all components.

Only industrial quality components were used for testing, with seal materials used according to the component manufacturer’s decision. These are based on general experiences and recommendations, usually intended for the use of hydraulic mineral oil. Let us mention once again that one of the most important goals of the development of the new ionic hydraulic fluids was the direct replacement of the oil for the ionic hydraulic fluid, without any changes to the equipment. The seal materials used in the tested components are listed in Table 3.

The hydraulic cylinder was not an off-the-shelf component like all the others but was purpose-built for testing purposes. For this reason, we had a free choice regarding the use of the seal material. Thus, based on the results of stationary thermal testing (Section 2.1), as well as on suitability for use as a dynamic seal, and based on the materials of the seals, we decided to manufacture seals from FKM. This material is also already present in other components.

### 3.3. Seal Compatibility Results in a Long-Term Durability Test

Based on the results stated in Section 2.1, as well as the recommendations and practical experience of seal manufacturers and hydraulic cylinder repairers, FKM and NBR are the most suitable materials for dynamic seals, although they did not prove to be the best in standard static testing with thermal loading. According to the results of the standardized static compatibility test, the materials POM, EPDM, and PTFE turned out to be better (i.e., smaller changes in terms of swelling, shrinking, and hardness), but due to their hardness, they are more suitable for use as guide rings than for elastic dynamically loaded seals. According to the static thermal loading of the seals, the use of FKM material is the best choice. Therefore, the dynamic seals of the hydraulic cylinder were made of this material for durability testing purposes.

As a first baseline, a long-term durability test was carried out with mineral oil, as a known fluid with expected behavior. In this way, the operation of the entire test device was also tested, including the acquisition and log of all signals. After this test, all key components were replaced with new equivalent ones (starpoint: a pre-selection process of components based on standardized measurements; and replacement of all hydraulic cylinder seals), and the entire system was flushed in several iterations (repetitions) before the HIL test, with measurement of the remaining amount of oil after each flushing.

**Figure 4:**
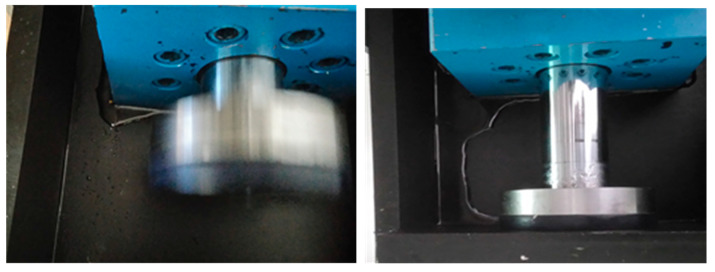
Start of leakage (**left**) and more extensive leakage—cylinder failure (**right**).

This was followed by a durability test with the same seal material and with HIL as the hydraulic fluid. However, after approx. 70,000 cycles of loading the hydraulic cylinder and the entire device, the first signs of leakage appeared, and, after approx. 100,000, the leakage was already quite noticeable. The test was stopped due to significant leakage. Figure 4 shows the first signs of leakage and the condition when the test was stopped.

**Figure 5:**
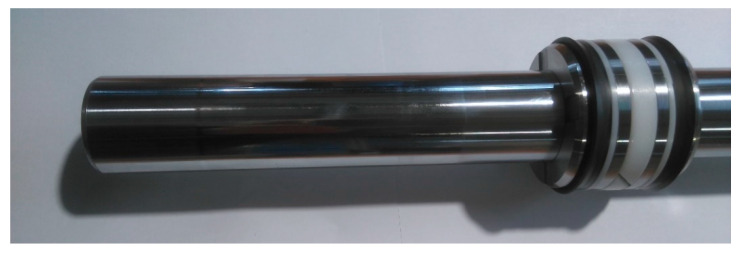
Worn seals on the piston of the test cylinder, and visible signs of wear on the piston rod as a result.

When disassembling the cylinder and inspecting the condition of the seals and sliding surfaces, it was found that the sealing edges of the seals showed signs of significant wear. Figure 5 shows the FKM seals on the cylinder piston and piston rod that have worn out (and become degraded in the sense of material property change) due to contact with the ionic hydraulic fluid during operation under real operating conditions.

The testing continued after replacing the piston seals and wiper made of FKM material with NBR, as the second-best material variant based on the static thermal test. The continuation of the test to the intended end, 1,000,000 cycles, was stable and without leaking problems. NBR seals were incorporated in all subsequent industrial applications using HIL B2002a^TM^, both for dynamic sealing, as in the case of hydraulic cylinders, and as static seals for all other components.

## 4. Discussion

For completely new types of hydraulic fluids, such as high-tech ionic hydraulic fluids, with completely different formulations than, e.g., classic hydraulic mineral oil, there are no Standards and standardized procedures for testing individual physicochemical properties. In such cases, we can only rely on the procedures and Standards that apply to classical, known fluids; in hydraulic drive control technology, these are usually mineral-based oils. As a result, there may be differences between the results obtained with Standard test procedures and the findings obtained during operation under real operating conditions. This type of issue also concerns the very important aspect of the compatibility of seal materials in hydraulic components.

In the presented example, the compatibility of the most-used seal materials with the high-tech ionic hydraulic fluid HIL B2002a^TM^ was tested, which is already established on hydraulic systems that operate in demanding operating conditions. The compatibility of the materials was checked using two different methods.

As a starting point for choosing the most suitable material for the seals in the case of using the mentioned ionic hydraulic fluid, a standardized static test was used, with different long phases of thermal loading of the seals immersed in the tested fluid. Before the test and after each phase of the test, the changes in the material that are important for the seal were measured: the swelling and shrinkage of the seal, as well as the change in the hardness of the seal. The compatibility of the fluid with a certain material was assessed, based on the comparison of the changes with the limit values that apply to mineral oils. In our case, following this procedure, FKM proved to be the best seal material for use with the ionic hydraulic fluid.

Although the Standard compatibility test is based on extensive measurements of seal changes, it does not yet provide the same results under real operating conditions. In cases where it is not a known fluid, deviations between the results can occur quickly, which can also lead to catastrophic consequences.

This was also proven in the case under consideration, when a long-term durability test of the entire hydraulic system was carried out with the new fluid under real operating conditions, with the focus also on the seals. In this test, the FKM material did not prove to be the most suitable, as the dynamic loading of the seals led quickly to an ever-increasing leakage of the hydraulic cylinder and the resulting failure. After replacing the material of the seals with NBR material and continuing the test as well during all the repeated tests, in the following industrial applications, there were no problems with NBR.

The results presented are a good starting point when deciding on a suitable seal material, in this case, HIL B2022a^TM^. The procedures presented must be repeated in the case of another type of ionic hydraulic fluid.

## Figures and Tables

**Table 1:** Compatibility testing results of seals’ materials with HIL B2002a^TM^ and hydraulic mineral-based oil HLP Type.

Material	Fluid	Change in [%] after 70 h	Change in [%] after 250 h	Change in [%] after 500 h
		Reference for oil: Volume swell: 15 Shrinkage: −3 Hardness: ±7	Reference for oil: Volume swell: 15 Shrinkage: −4 Hardness: ±8	Reference for oil: Volume swell: 20 Shrinkage: −4 Hardness: ±10
FKM	Hydraulic mineral-based oil HLP	Volume swell: 8 Shrinkage: 0 Hardness: −2	Volume swell: 33 Shrinkage: −19 Hardness: −2	Volume swell: 42 Shrinkage: −24 Hardness: −3
	HIL B2002a^TM^	Volume swell: 0 Shrinkage: −6 Hardness: −5	Volume swell: 0 Shrinkage: −6 Hardness: −9	Volume swell: 0 Shrinkage: −6 Hardness: −10
POM	Hydraulic mineral-based oil HLP	Volume swell: 7 Shrinkage: 0 Hardness: −2	Volume swell: 0 Shrinkage: 0 Hardness: −2	Volume swell: 0 Shrinkage: 0 Hardness: 0
	HIL B2002a^TM^	Volume swell: 0 Shrinkage: 0 Hardness: −1	Volume swell: 0 Shrinkage: 0 Hardness: −1	Volume swell: 0 Shrinkage: 0 Hardness change: −1
NBR	Hydraulic mineral-based oil HLP	Volume swell: 14 Shrinkage: −4 Hardness: 0	Volume swell: 14 Shrinkage: −8 Hardness: 0	Volume swell: 14 Shrinkage: −8 Hardness: 0
	HIL B2002a^TM^	Volume swell: 13 Shrinkage: −6 Hardness: 1	Volume swell: 20 Shrinkage: −11 Hardness: 1	Volume swell: 20 Shrinkage: −6 Hardness: 3
EPDM	Hydraulic mineral-based oil HLP	Volume swell: 24 Shrinkage: −33 Hardness: −11	Volume swell: 29 Shrinkage: −45 Hardness: −11	Volume swell: 35 Shrinkage: −48 Hardness: −15
	HIL B2002a^TM^	Volume swell: 6 Shrinkage: 0 Hardness: +1	Volume swell: 6 Shrinkage: −6 Hardness: 0	Volume swell: 0 Shrinkage: −6 Hardness: 0
MVQ	Hydraulic mineral-based oil HLP	Volume swell: 5 Shrinkage: 0 Hardness: −2	Volume swell: 5 Shrinkage: −5 Hardness: −5	Volume swell: 5 Shrinkage: 0 Hardness: −2
	HIL B2002a^TM^	Volume swell: 12 Shrinkage: −8 Hardness: +2	Volume swell: 15 Shrinkage: −17 Hardness: +2	Volume swell: 12 Shrinkage: −14 Hardness: 0
PTFE I	Hydraulic mineral-based oil HLP	Volume swell: 0 Shrinkage: 0 Hardness: −2	Volume swell: 0 Shrinkage: 0 Hardness: −2	Volume swell: 0 Shrinkage: 0 Hardness: −1
	HIL B2002a^TM^	Volume swell: 0 Shrinkage: 0 Hardness: −2	Volume swell: 0 Shrinkage: 0 Hardness: −2	Volume swell: 0 Shrinkage: 0 Hardness: −1

**Table 2:** Color change in the FKM material during stationary testing for compatibility with HIL B2002a^TM^.

Testing Time [h]	Before Test Start	After 70 h	After 250 h	After 500 h
Color change in FKM seal material	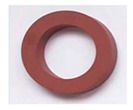	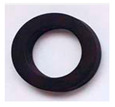	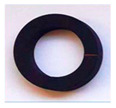	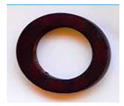

**Table 3:** Materials used in the tested components.

Component	Seal Material
Hydraulic gear pump	NBR (optional NBR and FKM)
Hydraulic proportional valve	NBR
Hydraulic cooler	NBR
Hydraulic fittings and couplings	FKM
Hydraulic filters’ housing	NBR
Hydraulic cylinder dynamic seals	FKM (based on compatibility test results)
Hydraulic cylinder wear rings	PTFE

## Data Availability

The original contributions presented in the study are included in the article; further inquiries can be directed to the corresponding author.
